# MIF coordinates the cell cycle with DNA damage checkpoints. Lessons from knockout mouse models

**DOI:** 10.1186/1747-1028-2-22

**Published:** 2007-07-19

**Authors:** Günter Fingerle-Rowson, Oleksi Petrenko

**Affiliations:** 1 University Hospital Cologne, Clinic I of Internal Medicine, Dept. of Hematology and Oncology, LFI, Level 4, Room 704, Kerpenerstr. 62, 50924 Cologne, Germany; 2 Department of Pathology, State University of New York at Stony Brook, Stony Brook, NY 11794, USA

## Abstract

Macrophage migration inhibitory factor (MIF) is a ubiquitously expressed pro-inflammatory mediator that has also been implicated in the process of oncogenic transformation and tumor progression. We used a genetic approach to show that deletion of the MIF gene in mice has several major consequences for the proliferative and transforming properties of cells. MIF-deficient cells exhibit increased resistance to oncogenic transformation. The transformation defects associated with MIF deficiency can be overcome through concomitant inactivation of the p53 and Rb/E2F tumor suppressor pathways. We have produced compelling evidence that the effects of MIF on cell survival and tumorigenesis are mediated through overlapping pathways, wherein MIF and p53 functionally antagonize each other in the cell. However, the involvement of MIF in p53 function is secondary to p53-independent mechanisms controlling protein stability, DNA damage checkpoints, and the integrity of the genome. Given the broad spectrum of cell types that normally express MIF and its elevated levels at sites of chronic inflammation, this pathway may be generic for many early stage tumors.

## Background

Chronic inflammation and neoplastic transformation are closely associated, since it is well established that an increased cancer risk exists in chronically inflamed tissues. Classic examples include ulcerative colitis-associated colorectal cancer [1,2], viral hepatitis-associated hepatocellular carcinoma [3], and Helicobacter pylori-associated gastric cancer [4]. Another example is provided by MALT lymphoma, where chronic infection causes persistent B-cell activation culminating in chromosomal rearrangements that lead to cancer [5]. It is thought that inflammation stimulates the formation of tumors through a mechanism involving the microenvironment and surrounding inflammatory cells [6,7]. However, the precise molecular links between inflammation and tumor development are not fully understood. Macrophage migration inhibitory factor (MIF) is a ubiquitously expressed pro-inflammatory mediator that has also been implicated in the process of oncogenic transformation and tumor progression [8]. Recent insights into the mechanistic basis of MIF action show that its interaction with Jab1/CSN5 is of crucial importance for the proper functioning of DNA damage response pathways [9]. By interfering with Jab1/CSN5, MIF controls the neddylation status of cullins and thereby the activity of SCF ubiquitin ligases. Here, we discuss the mechanisms by which MIF affects the ubiquitin-proteasome system, and how this impacts on the integrity of the genome and on cancer.

## Discussion

### MIF serves as a link between inflammation and cancer

Macrophage migration inhibitory factor was originally identified for its ability to inhibit the random migration of macrophages *in vitro *[10,11]. Subsequent work defined MIF as a potent cytokine with mitogenic and pro-inflammatory functions [12]. Recent efforts to identify a cellular surface receptor for MIF showed that the CD74/CD44 receptor complex mediates binding of extracellular MIF [13,14]. However, the process by which extracellular MIF may exert its effect on target cells is still poorly understood [15].

MIF is remarkably well conserved, and its homologues are encoded in evolutionarily divergent species, including different vertebrates, worms, insects and plants [16]. For a cytokine, MIF is unusual, since it is abundantly expressed by various cell types [12] and stored within the cytoplasm [17]. Early evidence suggesting a role for MIF in cell growth and/or differentiation came from the observations of its expression in developing mouse embryos. The MIF gene is expressed at early embryonic stages prior to implantation [18]. At mid-gestation, MIF's expression pattern parallels tissue specification and organogenesis [19,20]. At later developmental stages, MIF expression is broadly associated with cellular differentiation [21]. However, MIF appears to be dispensable for normal development, because MIF-null mice reproduce and grow normally [22,23].

Traditionally, the major focus of MIF research has been on its role as a pro-inflammatory mediator within the immune system. Recent studies, however, showed that MIF's functional repertoire is not limited to the immune response, but also extends to the regulation of apoptosis and malignant transformation. MIF overexpression has been observed in various human cancer tissues, including colorectal, breast, lung, bladder and prostate cancer [24-28]. Genetic studies demonstrated that MIF promotes B-cell lymphomagenesis and intestinal tumorigenesis in mice [24,29]. Importantly, MIF overexpression in several cell types confers resistance to apoptosis through interference with the activity of the p53 tumor suppressor [30]. Conversely, when MIF is lost, cell survival and functions are compromised in a p53-dependent manner [23,31]. Inhibition of MIF expression also phenocopies loss of hypoxia inducing factor-1α (HIF-1α), a well established target of p53 regulation, and induces premature senescence [32]. It was hypothesized that upregulation of MIF at sites of chronic inflammation might impair p53-dependent cellular responses towards DNA damage and inappropriate proliferation and, thus, promote the accumulation of oncogenic mutations [30].

In agreement with this, we recently showed that in a mouse model of Burkitt's lymphoma (Eμ-Myc transgenic mouse), loss of MIF expression coincides with the induction of a p53-dependent proliferative block, which profoundly affects normal B-cell development [29]. Moreover, inhibited S-phase progression and subsequent differentiation block are at the root of the predisposition of MIF-deficient B-cells to undergo spontaneous p53-dependent apoptosis. Accordingly, almost all lymphomas that arise in MIF-deficient Eμ-Myc mice can be accounted for by mutations within the ARF-p53 axis, indicating that the p53 pathway is the main determinant for tumor suppression in this model system [29].

**Figure 1:**
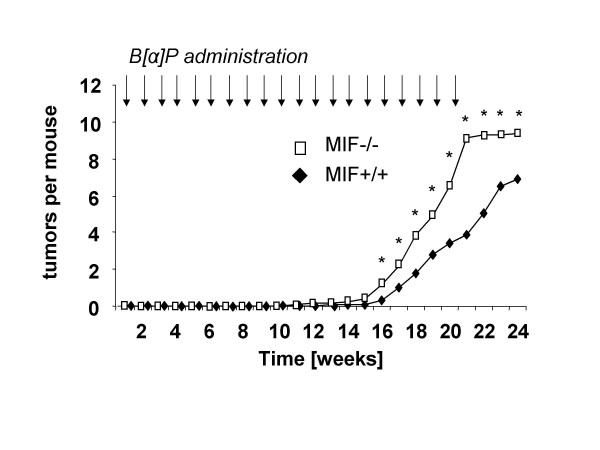
**MIF loss promotes skin tumorigenesis**. 6–10 week old male MIF^-/- ^or MIF^+/+ ^C57Bl/6 mice (n = 20 per group) were treated with 200 μg of the carcinogen benzo[α]pyrene (B[α]P) in 100 μl acetone topically on their backs once per week for 20 weeks. Skin tumors started to appear after week 14, and increased in number during the course of B[α]P treatment. MIF^-/- ^mice developed nearly twice as many tumors per mouse as MIF^+/+ ^controls. Based on histological evaluation by a blinded pathologist, this primarily reflected an increase of non-invasive tumors. By contrast, the number of invasive tumors was similar between the genotypes. Likewise, we found no significant difference with respect to tumor size or vascularization. * = statistically significant with p < 0.01 in a Student's t-test.

However, several observations suggest that the functional role of MIF in tumorigenesis is more complex than previously appreciated. In contrast to the notion of MIF as tumor promoter, two reports have indicated that aberrantly low levels of MIF in human tumors could also correlate with poor clinical prognosis and that subcellular compartimentalization of MIF may likewise be relevant [33,34]. Our own evidence from chemical one-stage skin carcinogenesis experiments revealed that deletion of the MIF gene may lead to increased rates of tumor formation in mice (Fig. 1). These findings are also supported by recent experiments using MIF-knockout mice in a p53-null background, which showed that MIF deficiency leads to a shift in the tumor spectrum: while the expected high frequency of T-cell lymphomas and fibrosarcomas was reduced upon MIF deficiency, the frequency of B-cell lymphomas and carcinomas was strongly increased. Moreover, the shift in the tumor spectrum led to decreased survival of MIF-/-p53-/- mice compared to the p53-/- controls [9]. Thus, MIF is unlikely to have sufficient prognostic value when used in isolation from other possible mutations, particularly those affecting p53.

### MIF interacts with Jab1/CSN5

Progression through the cell cycle depends on timely activation of cyclin-dependent kinases (CDKs), which act in conjunction with their corresponding cyclins. The protein levels of cyclins, CDK inhibitors and other major regulatory proteins are quantitatively controlled by the ubiquitin-proteasome system (UPS). Which protein is ubiquitylated and subsequently degraded relies on recognition by two principal E3 ubiquitin ligases, the anaphase-promoting/cyclosome complex (APC/C) and the Skp1-Cullin1-F-box (SCF) complex. These protein complexes appear to fulfill related but nonetheless distinct functions in the regulation of the cell cycle, as SCFs are active from late G1 to early M-phase, whereas the APC/C is activated from mid M-phase to the end of G1-phase [35]. The SCF complex has recently taken center stage in regulatory biology because it links extra- and intracellular signals to destruction of various proteins by the proteasome [36,37]. Thus, SCFs target many key proteins involved in the control of normal cell division, such as cyclin E, c-Jun, c-Myc, p21, p27, β-catenin and Notch [38-44]. Recent work shows that the SCF complex is also a central effector of DNA damage and repair pathways acting in the S- and G2M-phases of the cell cycle [45,46]. Therefore, it is not surprising that SCF is frequently a target of genetic alteration in cancer [37], and that deregulated SCF activity greatly promotes cancer development [47].

The SCF complex is made of four components, wherein Rbx1, Cul1 (scaffold protein) and Skp1 (adaptor protein) are invariable subunits. The fourth component, the F-box protein, serves as a substrate recognition unit. Given the variety of F-box proteins that can be recruited to the SCF complex (more than 70 F-box proteins have been identified in humans), the functional diversity of the SCF complex appears tantalizing [48]. The activity of the catalytic core of SCF, which is formed from the Rbx1 and Cul1 subunits, is stimulated by the attachment of ubiquitin-like protein Nedd8 to conserved lysines in the cullin-homology domain of Cul1 [49]. This step invokes recruitment of E2 enzymes and thus promotes assembly of an active SCF complex. Conversely, deneddylation of cullins through the CSN/COP9 signalosome, with its Jab1/CSN5 subunit directly cleaving off Nedd8, decreases E2 recruitment [50,51]. In addition, CSN recruits a deubiquitylase, Ubp12, which counteracts the intrinsic ubiquitin-polymerizing activity of SCF [52] Furthermore, deneddylated cullins are sequestered by inhibitory Cand1 [53,54]. It was proposed that SCF activity is sustained by dynamic cycles of assembly and disassembly, where both Cand1 and CSN play an essential negative role [55].

**Figure 2:**
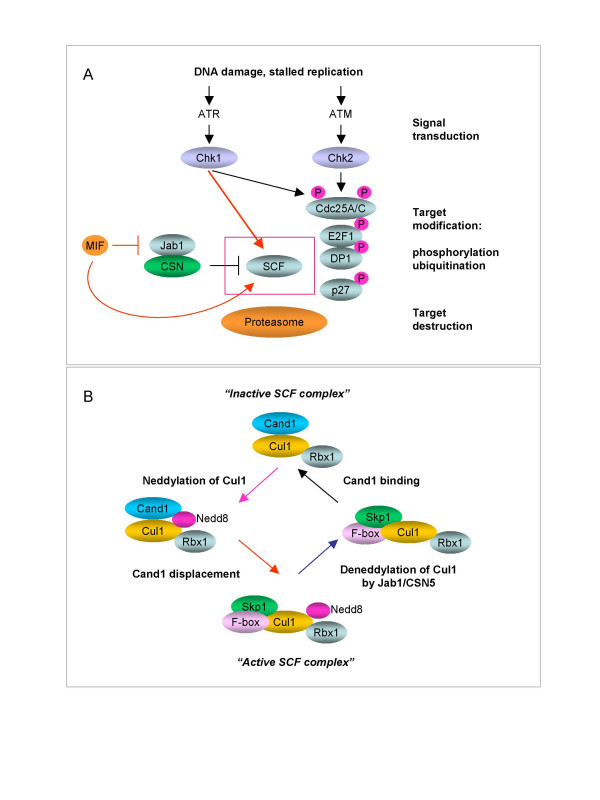
**SCF activity is sustained by dynamic cycles of assembly and disassembly**. **(A)**. DNA damage checkpoint pathways feed into the proteolytic degradation of key cell cycle regulators, mediated by SCF, to stop the cell cycle. The scheme indicates the elements that make up signal transducers (ATM, ATR, Chk1, Chk2) and effectors (SCF, CSN and 26S proteasome). MIF binds to Jab1/CSN5 and prevents it from interacting with proteins targeted by CSN, notably the Cullins. **(B)**. Multi-subunit structure of the SCF class of E3 ubiquitin ligases. All SCFs consist of Cullins, Skp1, Rbx1 and F-box proteins which associate to form an enzymatically active complex. The posttranslational modification of Cullin (Cul1) by Nedd8 renders SCF active, though unstable. The removal of Nedd8 from Cullin is catalyzed by Jab1/CSN5. Following deneddylation of Cullins, Skp1 and F-box proteins are replaced by the inhibitory protein Cand1.

Remarkably, a search for intracellular MIF-binding partners by the yeast two-hybrid system yielded Jab1/CSN5 as potential candidate [56]. CSN5 is a component of the COP9/CSN signalosome, a multiprotein complex that plays essential roles in differentiation and morphogenesis [57,58]. Not surprisingly, deletion of individual subunits of the CSN complex is embryonically lethal [59,60]. Also Jab1-null embryos die soon after implantation due to impaired proliferation and accelerated apoptosis, and these defects have been attributed in part to the accumulation and/or impaired degradation of p53, cyclin E, and p27 [61]. Importantly, Jab1 possesses an intrinsic metalloprotease activity, which as mentioned above, targets SCF complexes and deconjugates Nedd8 from the cullins [51,58]. Whereas Jab1 recycles neddylated cullins into more stable unneddylated forms [62], the CSN signalosome can further stabilize the SCF by preventing the autoubiquitination of substrate-recruiting F-box proteins [63,64]. However, deneddylated cullins are open to interaction with the inhibitory Cand1, which has the capacity to displace both Skp1 and F-box proteins [53,54]. Therefore, unbalanced activity of Jab1/CSN5 can directly or indirectly block ubiquitin-dependent proteolysis [65]. Conversely, MIF binds to Jab1/CSN5 and prevents it from interacting with proteins targeted by the CSN signalosome [66] (Fig. 2). Our recent study showed that this negative regulation of Jab1/CSN5 by MIF is a physiologic requirement to sustain optimal composition and activity of SCF ubiquitin ligases [9].

### MIF coordinates the cell cycle with DNA damage checkpoints

Cells continuously encounter DNA damage caused either by replication errors at replication forks or by extracellular noxae such as ultraviolet and ionizing irradiation. Failure to properly repair DNA can lead to various disorders, including enhanced rates of tumor development [67,68]. To protect against such insults to the genome, eukaryotic organisms have evolved an elaborate signalling network of p53-dependent and p53-independent checkpoints. Once the integrity of the genome has been compromised, checkpoint proteins either prevent or delay initiation of the next cell cycle phase. Notably, DNA damage checkpoint kinases (ATM, ATR, Chk1, Chk2) inhibit the Cdk machinery, the normal function of which is to coordinate DNA replication and partitioning of the chromosomes [69] (Fig. 2). The DNA damage response also causes induction of DNA repair functions, such that cells with modest damage may survive. However, cells with more severe damage are induced to undergo apoptosis. Mutations affecting DNA damage pathways allow cell proliferation under conditions of replication stress [68,70]. Therefore, the DNA damage response is subject to tight control and is regulated at the level of gene expression, protein phosphorylation, protein stabilization or degradation. Cancer development frequently selects for loss of p53 function and hence for loss of the G1 checkpoint [71]. Mutations compromising DNA damage checkpoints are also oncogenic [67,70], although they are expected to activate p53.

One of the key functional targets of DNA-damage checkpoint regulation is the Cdc25 protein phosphatase family, which controls cell cycle progression by regulating the activity of Cdk2 and Cdk1 kinase complexes [68]. We found that in MIF-deficient mice, Chk1/Chk2-regulated checkpoints are uncoupled from proteasomal degradation of Cdc25A under conditions of DNA damage [9]. The loss of MIF produced similar dysregulation in the proper degradation of several other cell cycle regulators such as Cyclin A2, p27, E2F1 and DP1 [9]. Because MIF binds to Jab1/CSN5 and prevents it from interacting with proteins targeted by CSN, notably the cullins, it appears that absence of this interaction can lead to inappropriate deneddylating activity of Jab1/CSN5 and therefore to the ensuing dysfunction of the SCF. Importantly, we showed that DNA damage induces coordinate activity of the G2M checkpoint and Cul1-containing SCF complexes. Moreover, Chk1 delivers a signal that not only marks proteins for degradation but also activates the SCF (Fig 2). These data emphasize the importance of downstream effectors of checkpoint pathways that execute the cell division shut-off program, namely the SCF. In essence, our results imply that the G2M checkpoint and the SCF complex form a functional unit to stop cell cycle progression after DNA damage [9]. These data also provide an attractive mechanism explaining the MIF-p53 relationship and the role of MIF in tumor development. Given that MIF plays a key role in the regulation of Cdc25A, Cdk2 and E2F1/DP1 complexes, which can all induce a strong p53-dependent antiproliferative response, our data suggest that the involvement of MIF in p53 function is secondary to p53-independent mechanisms controlling protein stability, checkpoint regulation, and the integrity of the genome. While the loss of MIF expression induces a p53-dependent proliferative block [23,29], concomitant loss of p53 rescues these growth defects, but it comes at the price of increased tumorigenesis [9]. Accordingly, the tumor phenotype of MIF/p53 compound mutant mice entails defects in the checkpoint response and DNA repair process [9].

## Conclusion

Until recently, MIF was primarily viewed as a tumor promoter through its signaling activities which inhibit p53-dependent apoptosis [8,15]. Nowadays, it is well established that chronic inflammation increases the risk of cancer. The important role of MIF in inflammation indeed suggests that it is not only promoting existing tumors, but also likely of great relevance for the initiation of cancer by chronic inflammatory processes. MIF's mechanism of action reinforces the functional connection between DNA damage, genomic instability and cancer. By viewing MIF as a component of a molecular machine that governs the cell cycle via activity of the SCF complex, it becomes clear that MIF-specific effects on tumor initiation and/or development will inevitably depend on a variety of parameters, including tissue-specific and microenvironmental aspects. Furthermore, it is conceivable that a better understanding of MIF biology will help to design novel strategies for cancer treatment. Clinically relevant examples are tumors with loss of p53 function, which constitute a substantial clinical problem due to their poor response to chemotherapy or irradiation [72]. Indeed, the G2M response elicits signals that can trigger not only growth arrest or apoptosis, but also direct activation of DNA repair networks [73]. Therefore, cancer cells must retain sufficient G2M checkpoint function in order to survive adverse conditions that could further destabilize the genome, causing mitotic catastrophe and cell death. Accordingly, severe disabling of G2M signaling is viewed as a possible anticancer strategy [67,68,74]. It is believed that inactivation of G2M checkpoint function would favor tumor cell death by enhancing the cytotoxic effect of chemotherapeutic reagents. Evidence supporting the role of MIF in the G2M response suggests that it could be an attractive target for therapeutic intervention. Specifically, targeting the MIF-Jab1/CSN5-SCF interaction may have important implications, since deregulated SCF plays a fundamental role in the development and survival of many types of human malignancies.

## Abbreviations

MIF, macrophage migration inhibitory factor

Jab1, c-jun activating binding protein-1

SCF, skp-cullin-F-Box protein complex

Ubc12, ubiquitin-conjugating enzyme-12

## Competing interests

The author(s) declare that they have no competing interests.

## Authors' contributions

GFR and OP both drafted the manuscript and designed the figures. The authors read and approved the final manuscript.
